# Primary fourth ventricular B-cell lymphoma in an immunocompetent patient 

**DOI:** 10.5414/NP300658

**Published:** 2013-08-07

**Authors:** Andrew J. Fabiano, Susanna A. Syriac, Robert A. Fenstermaker, Jingxin Qiu

**Affiliations:** 1 Department of Neurosurgery, Roswell Park Cancer Institute,; 2 Department of Neurosurgery, School of Medicine and Biomedical Sciences, University at Buffalo, State University of New York, and; 3 Department of Pathology and Laboratory Medicine, Roswell Park Cancer Institute, Buffalo, NY, USA

**Keywords:** B cell, fourth ventricle, primary central nervous system lymphoma

## Abstract

Letter to the Editor.

Sir, – Primary central nervous system lymphoma (PCNSL) is a malignant lymphoma that arises within the parenchyma of the brain or spinal cord. The incidence of PCNSL has been rising over the last few decades, particularly in immunologically compromised patients with acquired immunodeficiency syndrome [1, 2, 3, 4]. It is a rare diagnosis in the immunocompetent patient [5, 6, 7]. Most PCNSLs are diffuse, large B-cell lymphomas with a different biological behavior, management and prognosis than systemic diffuse large B-cell lymphomas [8]. The majority of these lesions are located in the cerebral cortex. The involvement of other brain regions (cerebellum, brainstem or spinal cord) is usually associated with multifocal disease [2]. The authors present a case of a PCNSL located in the fourth ventricle of an immunocompetent patient. 

The patient is a 60-year-old woman with a history of infiltrating ductal adenocarcinoma of the left breast underwent a left partial mastectomy and left sentinel node biopsy. She presented with the new onset of diplopia 3 months later. A magnetic resonance (MR) image of the brain demonstrated an ovoid fourth ventricular mass that was homogeneously enhancing with contrast material and extended from the left lateral recess of the fourth ventricle to the adjacent paramedian cerebellum without obstructive hydrocephalus (Figure 1). Computed tomographic (CT) scans of the chest, abdomen and pelvis with and without contrast material were within normal limits. 

The patient underwent a posterior fossa craniotomy for removal of the fourth ventricular tumor. Pathologic examination of the tumor revealed discohesive, large, pleomorphic cells that were strongly immunoreactive for CD45, CD20 and CD10 proteins, with a Ki-67 proliferation index of nearly 100% (Figure 2). Tumor cells were weakly immunoreactive for B-cell lymphoma 2 (bcl-2), B-cell lymphoma 6 (bcl-6), and paired box protein (PAX-5), had rare reactivity for multiple myeloma oncogene 1 (MUM-1) (less than 30% tumor cells), and were negative for CD34, lysozyme, CD3, myeloperoxidase, glial fibrillary acidic protein, synaptophysin, S-100 and EMA. This tumor lacked the angiocentric distribution of lymphoma cells that is classically described for intraparenchymal PCNSLs [9]. There was demarcation of the main tumor mass from the adjacent brain tissue, which had a few scattered lymphoma cells present. In situ hybridization studies showed bcl-6 gene translocation, in the absence of bcl-2 and C-MYC gene translocations. A quantitative real-time polymerase chain reaction (PCR) study showed clonal immunoglobulin heavy locus (IgH) gene rearrangements. These findings confirmed the diagnosis of a diffuse large B-cell lymphoma (DLBCL) type of PCNSL. This patient had a serum complete blood count within normal limits and multiple bone marrow biopsies and cerebral spinal fluid specimens that were negative for lymphoma. Additional body CT scan, positron emission tomographic scan and bone scan did not show any evidence of adenopathy or metastatic breast cancer. She was placed on the DeAngelis chemotherapy protocol [10] and tolerated the protocol well. Six months postoperatively, she is clinically well with no sign of recurrence. 

Three cases of solitary PCNSL arising in the fourth ventricle have been previously reported [5, 6, 7]. The first case was a 17-year-old woman with a clinical presentation of meningitis, and the tumor was diagnosed post-mortem [7]. The second case was a 33-year-old woman with headaches and vertigo [5]. MR imaging revealed a homogeneous fourth ventricular B-cell lymphoma that was completely excised. The third case was a 69-year-old man with a clinical presentation of 6 weeks of intractable vomiting [6]. MR imaging showed a homogeneously enhancing mass in the caudal fourth ventricle. Surgical excision was performed, and pathological examination demonstrated a high-grade B-cell lymphoma. 

Our case, along with the other reported cases [5, 6, 7], showed that PCNSL can arise in rare instances from the fourth ventricle as a solitary mass lesion (Table 1). All four patients were immunologically competent, with ages ranging from 17 to 69 years. Clinical presentation involves symptoms secondary to cerebellar mass effect, including headaches, vertigo, vomiting and diplopia. These tumors are homogeneously enhancing on MR imaging and tend to exhibit an exophytic growth pattern into the fourth ventricle. Surgical excision of the tumor followed by chemotherapy has shown good response in 3 of the 4 patients. 

The origin of these solitary fourth ventricular PCNSLs remains uncertain. In our case, the main tumor mass was demarcated from adjacent brain tissue. There were only a few scattered lymphoma cells present in the adjacent brain tissue. The typical angiocentric infiltration pattern of the PCNSL [9] was not present in this tumor. The immunohistochemical studies showed that the tumor has a germinal center B-cell-like profile (bcl-2+/bcl-6+/CD10+), which is consistent with the typical PCNSL. However, the MUM-1 immunoreactivity was only focal and patchy (less than 30% of the tumor cells). This is different from the typical PCNSL, which has near 100% MUM-1 strong immunoreactivity [11]. 

In rare instances, PCNSL can occur as a solitary mass lesion in the fourth ventricle. Primary B-cell lymphoma should be included in the differential diagnosis of posterior fossa mass lesions, including lesions identified in immunocompetent patients. Given the immunohistologic differences between our specimen and the classic description of PCNSL tissue, additional studies are needed to further characterize these solitary fourth ventricular PCNSLs when more cases become available. 

## Acknowledgments 

This work was supported by Roswell Park Cancer Institute and National Cancer Institute (NCI) grant #P30 CA016056. The authors have no other financial relationships to disclose. We thank Paul H. Dressel, BFA, for assistance with preparation of the illustrations and Debra J. Zimmer, AAS CMA-A, for editorial assistance. 

**Table 1:** Summary of 4 cases of fourth ventricular primary central nervous system lymphoma reported in the literature.

Authors, year	Patient age/sex	Previous history	Clinical presentation	MR imaging findings	Other findings (including CT scan, blood test, bone marrow biopsy)	Surgical procedure	Diagnosis	Treatment	Follow-up
Werneck et al. 1977 [7]	17/F	N/A	Meningitis	N/A	N/A	N/A	primary CNS lymphoma	N/A	N/A
Haegelen et al. 2001 [5]	33/F	none	vertigo and headaches	homogeneous enhancing mass	negative	excision	large, high-grade B-cell lymphoma	chemotherapy and autologous stem-cell transplantation	7 months without recurrence
Hill et al. 2009 [6]	69/M	N/A	intractable vomiting, mild preceding nausea, anorexia, weight loss; no headache	homogeneous enhancing mass	negative	excision	high-grade B-cell lymphoma	chemotherapy	3 months without recurrence
This case	60/F	breast cancer	diplopia	homogeneous enhancing mass	negative	excision	diffuse, large, B-cell lymphoma	DeAngelis Protocol [10]	6 months without recurrence

CNS = central nervous system, CT = computed tomographic, F = female, M = male, mo = months, MR = magnetic resonance, N/A = not available.

**Figure 1.**
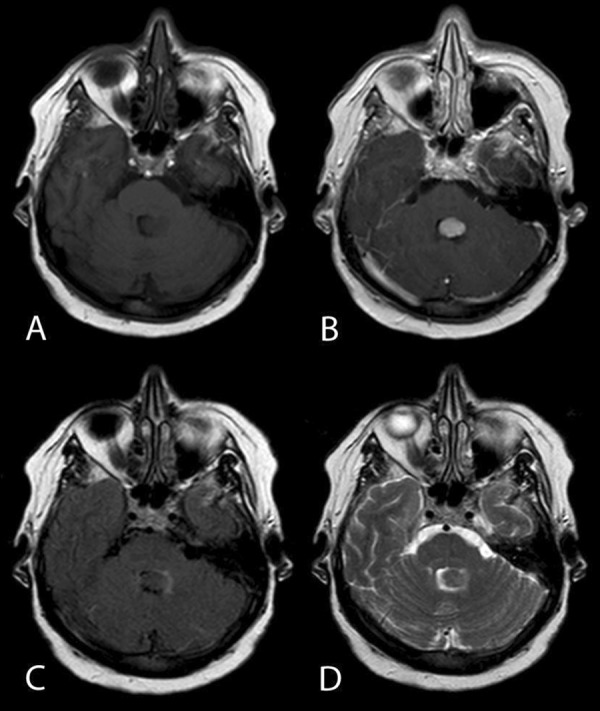
MR images obtained from a 60-year-old woman with diplopia. Axial T1-weighted images without (A) and with (B) contrast enhancement demonstrate a solitary, contrast-enhancing mass lesion within the fourth ventricle. The mass has an isointense signal to cortex on both fluid-attenuated inversion recovery (FLAIR) (C) and T2-weighted (D) pulse sequences.

**Figure 2.**
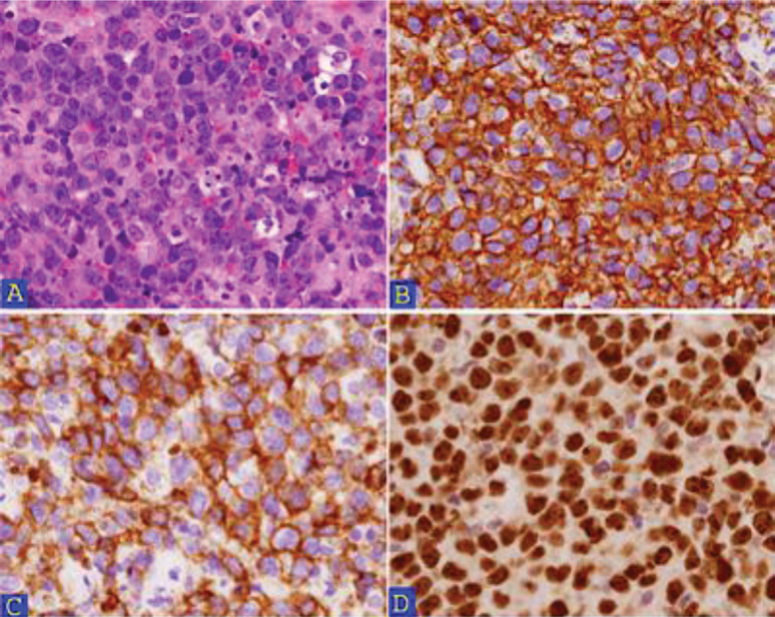
A: Hematoxylin-eosin staining of the PCNSL shows discohesive, large, pleomorphic cells with mitosis and apoptosis. Immunohistochemistry shows diffuse strong reactivity for CD20 (B) and CD10 (C). D: the Ki67 labeling index of the PCNSL is close to 100%.
